# Semantic enrichment of longitudinal clinical study data using the CDISC standards and the semantic statistics vocabularies

**DOI:** 10.1186/s13326-015-0012-6

**Published:** 2015-04-09

**Authors:** Hugo Leroux, Laurent Lefort

**Affiliations:** The Australian e-Health Research Centre, Digital Productivity Flagship, CSIRO, Level 5 - UQ Health Sciences Building 901/16, Brisbane, 4029 Queensland Australia; Digital Economy Program, Digital Productivity Flagship, CSIRO, Canberra, 2601 ACT Australia

**Keywords:** Ontology, Semantic enrichment, Longitudinal clinical study, RDF data cube, Medication mapping

## Abstract

**Background:**

There is an increasing recognition of the need for the data capture phase of clinical studies to be improved and for more effective sharing of clinical data. The Health Care and Life Sciences community has embraced semantic technologies to facilitate the integration of health data from electronic health records, clinical studies and pharmaceutical research. This paper explores the integration of clinical study data exchange standards and semantic statistic vocabularies to deliver clinical data as linked data in a format that is easier to enrich with links to complementary data sources and consume by a broad user base.

**Methods:**

We propose a Linked Clinical Data Cube (LCDC), which combines the strength of the RDF Data Cube and DDI-RDF vocabulary to enrich clinical data based on the CDISC standards. The CDISC standards provide the mechanisms for the data to be standardised, made more accessible and accountable whereas the RDF Data Cube and DDI-RDF vocabularies provide novel approaches to managing large volumes of heterogeneous linked data resources.

**Results:**

We validate our approach using a large-scale longitudinal clinical study into neurodegenerative diseases. This dataset, comprising more than 1600 variables clustered in 25 different sub-domains, has been fully converted into RDF forming one main data cube and one specialised cube for each sub-domain. One sub-domain, the Medications specialised cube, has been linked to relevant external vocabularies, such as the Australian Medicines Terminology and the ATC DDD taxonomy and DrugBank terminology. This provides new dimensions on which to query the data that promote the exploration of drug-drug and drug-disease interactions.

**Conclusions:**

This implementation highlights the effectiveness of the association of the semantic statistics vocabularies for the publication of large heterogeneous data sets as linked data and the integration of the semantic statistics vocabularies with the CDISC standards. In particular, it demonstrates the potential of the two vocabularies in overcoming the monolithic nature of the underlying model and improving the navigation and querying of the data from multiple angles to support richer data analysis of clinical study data. The forecasted benefits are more efficient use of clinicians’ time and the potential to facilitate cross-study analysis.

## Background

In the last decade, the Health Care and Life Sciences community and pharmaceutical industry have wholeheartedly adopted [1] clinical study data exchange technologies based on XML to capture clinical study data. This is largely due to the recent strategy [2] of the Food and Drug Administration (FDA) in promoting the Clinical Data Interchange Standards Consortium (CDISC) suite of standards to facilitate data submission and exchange. Furthermore, the move by EU and US regulating bodies to open access to clinical data [3,4] will also foster the adoption of tools supporting clinical data management standards, especially those that can easily be linked to methods and tools developed for Government Linked Data and Linked Science Data.

CDISC has developed a set of platform-independent data standards [5] for the collection and dissemination of clinical trial data. The CDISC Operational Data Model (ODM) is an XML format that facilitates the exchange of clinical data captured during a clinical study. ODM-based files contain the study data and the associated descriptions of the data items, their groupings into Case Report Forms (CRFs), which are electronic documents to record the study data, and the associated questions and code lists. Furthermore, the FDA has mandated the use of other CDISC standards in clinical studies. In particular the CDISC Study Data Tabulation Model (SDTM) is used to facilitate study metadata submissions and improve the accountability of the study data. The role of the CDISC Clinical Data Acquisition Standards Harmonization (CDASH) is to standardise the generation of CRFs for clinical studies. The implementation of the ODM, STDM and CDASH standards in Clinical Data Management Systems (CDMS) has enabled larger and more diverse longitudinal clinical research studies and increased the capability of users to exchange and combine data [6].

### Challenges relating to the cross-study analysis of clinical study data

A number of limitations relating to the reporting of results derived from current clinical trial endeavours were identified by van Valkenhoef et al. [7]. In particular, they stress that: “*current infrastructure is focused on text-based reports of single studies, whereas efficient evidence-based medicine requires the automated integration of multiple clinical trials from different information resources*” [7]. They specifically advocate for a comprehensive record of clinical trials to be made available in a machine understandable format that would improve the efficiency of evidence-based decision making but more importantly that decisions could then finally be explicitly linked back to the underlying data. Chief among their list of topics for future research directions are: (i) *the development of a comprehensive data model for clinical trials and their aggregate level results*; and (ii) *the development of a platform to share structured systematic review data sets*.

### Our contribution: semantic enrichment

This research builds upon existing work [8] to semantically enrich longitudinal clinical study data, based on the CDISC standards, using semantic statistic vocabularies, namely the RDF Data Cube and DDI-RDF vocabularies. We propose a Linked Clinical Data Cube, a set of modular data cubes that helps manage the multi-dimensional and multi-disciplinary nature of the clinical data. The RDF Data Cube vocabulary [9] is used to build multi-dimensional data cubes and supports flexible access to the data via thematic slices. The DDI-RDF Discovery vocabulary [10] is effective at encoding the study-specific data dictionary embedded in the CDISC ODM standard as linked data and helps in managing the link between the data cube variables and the data.

Our objective is to make the data captured within the Australian, Imaging, Biomarker and Lifestyle study of Ageing (AIBL) [11] seamlessly available to researchers who wish to engage in cross-domain analysis of the data. We achieve our goal by semantically enriching the data, when possible, with external data sources. Our approach is four-fold: *Phase 1: Integrating the CDISC ODM data model with the semantic statistic vocabularies.* We describe how the clinical data available in CDISC ODM can be mapped to the RDF Data Cube and DDI-RDF Discovery vocabulary to form the Linked Clinical Data Cube. *Phase 2: Splitting the data into modularised cubes.* We outline the design principles of splitting the data into more modularised and manageable groupings to provide alternative mechanisms for accessing and querying the data. The RDF Data Cube and DDI-RDF vocabularies are pivotal elements of our slicing strategy and of the URI scheme defined for our implementation. *Phase 3: Enriching the LCDC with the CDISC standards.* We discuss how useful the benefits of clinical study data to adhering to the CDISC CDASH and SDTM standards then elaborate on guidelines to classify the data into the broad categories. *Phase 4: Mapping the data to drug terminologies.* We demonstrate the utility of the LCDC by mapping the medications data derived from the AIBL study to selected online drug terminologies.

### The AIBL study

**Figure 1:**
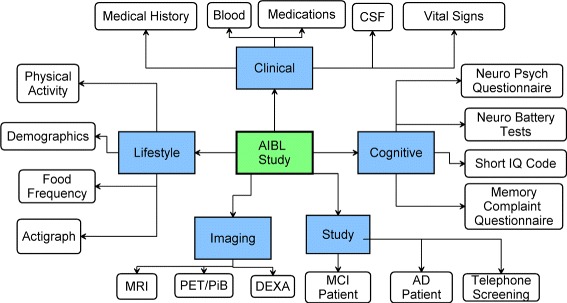
The Australian imaging biomarker and lifestyle study of ageing. Illustrates the logical organisation of the *AIBL* study. The *AIBL* study (depicted as a rectangle in light green with thick border) is split into the five domains (depicted as rectangles in light blue). Each domain is further categorised into sub-domains depicted by rounded rectangles.

AIBL is a prospective study of a large group (1112) of individuals residing in two Australian cities, Perth and Melbourne, aged over 60 years who are either classified as cognitively healthy, or meet clinical criteria for mild cognitive impairment or Alzheimer’s Disease and who have agreed to reassessment every 18 months. Assessment comprises extensive study of cognitive function, neuroimaging, blood biomarkers and lifestyle (diet and exercise) characteristics [11]. By combining these investigations in a prospective fashion, the AIBL study contributes to understanding the development and progression of Alzheimer’s Disease through the prodromal, preclinical and clinical stages of the disease [12]. It is vital for the clinical data to be reported at regular intervals as the study progresses. To facilitate this task, the study data is manually entered into the OpenClinica Clinical Data Management System (CDMS) by study staff [13]. Figure 1 describes the AIBL study with the five main categories and sub-categories.

OpenClinica [14] is an open-source CDMS for collecting and managing clinical data. The AIBL study data was successfully migrated to this platform in 2011 [13] and has been live since August 2011. OpenClinica supports the creation of customisable studies and the design of user-defined Case Report Forms (CRFs) using an Excel spreadsheet and adheres to the CDISC ODM standard. The data collected for the AIBL study covers multiple domains as shown in Figure 1. This dataset comprises more than 1600 variables clustered into 25 different sub-domains. The study has been split into five themes: *Study*, *Clinical*, *Cognitive*, *Imaging* and *Lifestyle*. The *Study* theme comprises administrative information that, for the most part, is not shared within the cube. Table 1 depicts the total number of instances for the various LCDC classes organised by theme. The total number of variables, in the table, is smaller than 1600 because the generation to RDF suppresses duplicates.

**Table 1:** **Number of instances for the LCDC classes organised by theme**

**Theme**	**Total**	**Obs.**	**Obs.**	**Subject**	**Sub-theme**	**Sub-theme**	**Variable**
		**group**		**section**	**series**	**slice**	
Clinical	1030430	4495	25210	1416	25	6452	506
Cognitive	761650	4612	9826	1415	19	4069	367
Imaging	58601	866	2136	365	12	941	59
Lifestyle	710594	4026	11953	1415	19	7360	391
Study	235566	5384	6218	1414	13	3292	155

### Article outline

In the remainder of this article, we outline an approach to semantically enrich clinical study data, in particular patient-reported medication usage, and facilitate their delivery to clinical researchers. In particular, we outline how the use of semantic statistics vocabularies is effective at organising the data into a LCDC. We also elaborate on the approach taken to categorise the AIBL data set into CDISC CDASH and SDTM domains and the work carried out to translate the CDISC standards into RDF. This leads into the discussion on the design principles for the LCDC and of the benefits of splitting the data into more modularised groupings.

## Methods

The LCDC [15] comprises one main cube and several specialised cubes, one for each domain within the study, that integrates the CDISC ODM data model with the RDF Data Cube and DDI-RDF vocabularies. We elaborate further on the rationale behind this integration below. The LCDC is designed around a set of cubes, slices, observation groups and observations and these are discussed further below. The ability to standardise the clinical data in order to facilitate cross-domain and, possibly, cross-study analysis of the data is one of the salient objectives of the LCDC. To this end, we describe how the study variables have been enriched by the CDISC CDASH and SDTM standards. Aside from providing a standardised representation to the study variables and grouping them along the various CDISC categories, this enrichment process allows for seamless substitution of variable names in the navigation and querying of the clinical study. Finally, we outline how the coupling of the study data with external resources - in this case drug terminologies - can be achieved within the LCDC and we elaborate on our process to implement a *linked medications data set* and how the patient-reported medication intake from the AIBL study has been mapped to this data set.

### Phase 1: Integrating the CDISC ODM data model with the semantic statistic vocabularies

Clinical study data is extracted in CDISC ODM format. The primary dimensions of the CDISC ODM data model are the Subject and Study Event of interest within the study. The additional dimensions, including the Study, Form, ItemGroup and Item, depend on the study domains and are specified by the data dictionary that defines the study. The strength of the RDF Data Cube is that the original structure of the CDISC ODM data model (Study-Subject-StudyEvent-Form-ItemGroup-Item) lends itself to be replicated in the generated cube with relative ease. A further contribution of the RDF Data Cube is that it can help overcome the monolithic nature of the ODM data model by facilitating the construction of multi-dimensional cubes that offer access points to the data via thematic slices. The LCDC is organised into one main cube and several specialised cubes corresponding to the various domains in the study.

**Figure 2:**
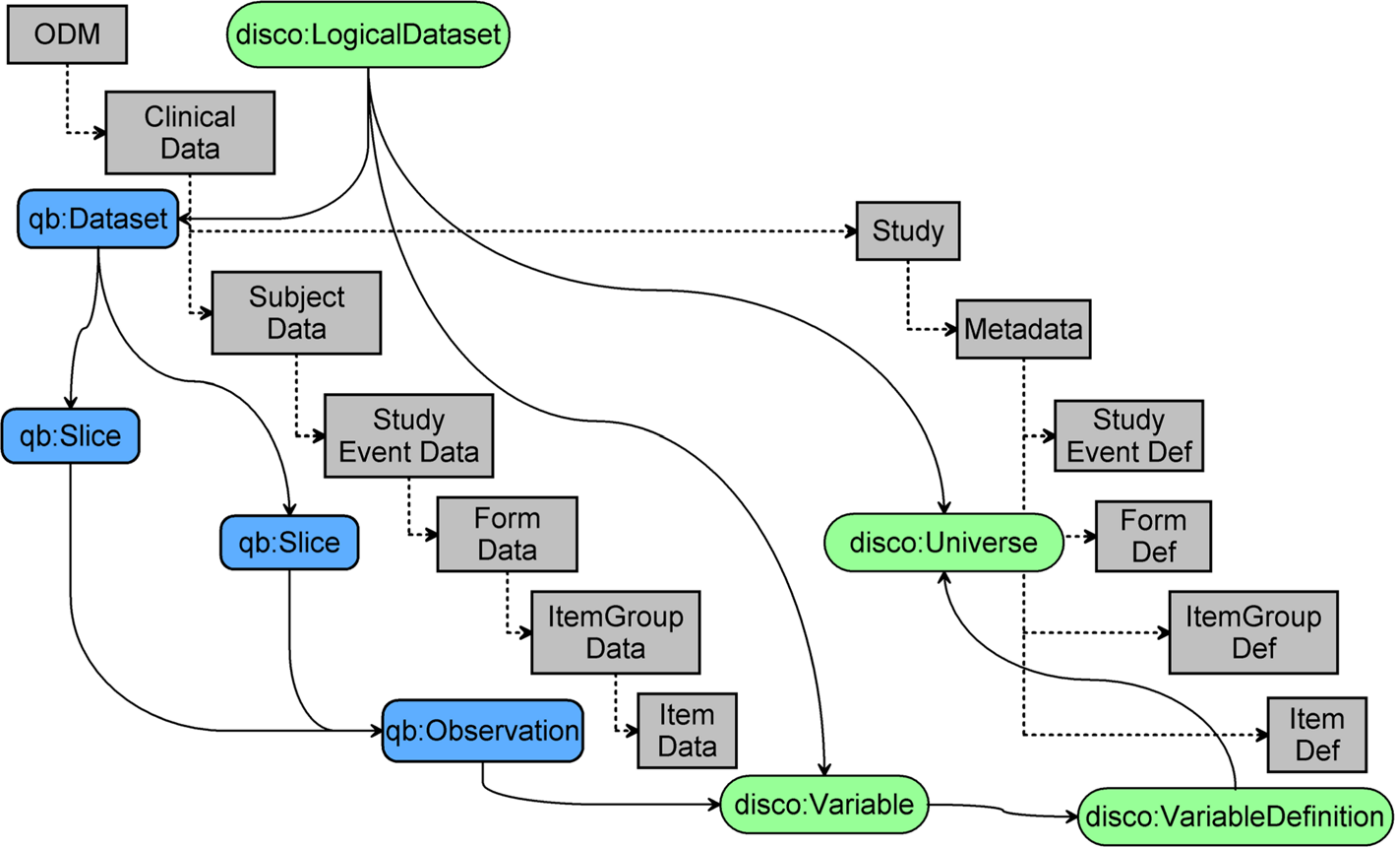
Mapping the CDISC ODM model to the data cube and DDI vocabularies. Illustrates how the original *CDISC ODM model* (depicted by rectangles in light gray) is overlaid with the *RDF Data Cube* (depicted by ellipses in green) and the *DDI-RDF* vocabularies (depicted by rounded rectangles in blue). The Data section, depicted on the left of the model, comprises a hierarchical structure whereby each level is fully contained within the preceding level. As the left side is more about structuring the clinical data, the Data section of the *CDISC ODM* model is more closely related to qb. The Clinical Data node is mapped to qb:Dataset while qb:Slice is used to split the Subject, Study Event and Form data nodes across the ODM hierarchy into slices, and the *Item Data* node is mapped to qb:Observation. The ODM node refers to the entire data set and is mapped to disco:LogicalDataset. The right side comprises the metadata section, which contains one Study node, which further comprises one MetaData node. The MetaData node contains a number of StudyEventDef, FormDef, ItemGroupDef and ItemDef nodes, one corresponding to each of the Subject, Study Event, Form, Item Group and Item data nodes defined in the Data section. The Metadata section shows how the variable definitions managed through disco matches ODM’s ItemDef while the grouping of variables via disco:Universe is applied at the FormDef level. Finally, Item Data is logically mapped to disco:Variable.

The RDF Data Cube model facilitates the grouping of subsets of observations, within the dataset, whereby all but one (or a small subset) of the dimensions are fixed. Furthermore, it supports alternative methods of accessing the data where the data is aggregated along other dimensions or along the same dimension in different order. The DDI-RDF Discovery vocabulary is used to consistently manage the study-specific data dictionary exported in CDISC ODM format enriched with CDISC metadata resources (CDASH and SDTM). These two vocabularies are supplemented by the Vocabulary of Interlinked Dataset (VoID). These allow the LCDC ontology to be defined with more generalised classes and properties, such as the disco:Universe, disco:Variable and disco:VariableDefinition [15] as depicted in Figure 2.

### Phase 2: Splitting the data into modularised cubes

The design of the LCDC is achieved in three steps. The first step involves splitting the dataset into smaller, more manageable specialised cubes. The second step is to define several slice hierarchies that offer multiple access options to the individual data records. The third step is to define a URI scheme that supports access to the cube at all levels of the slice hierarchy. These three steps are discussed below.

The LCDC defines three categories of slices. The *time-series* slices address the longitudinal nature of the study and organise the data into time-intervals and dated and non-dated time points. *Cross-section* slices adopt a subject-centric approach to the abstraction of the data along some important concepts such as gender, genotype and neurological classifications. The *Theme* slices categorise the data into the study domains and sub-domains (disco:Universe in DDI-RDF) and help link the main and specialised cubes together. This process enhances the navigation and querying of the data in the LCDC because we provide three direct links to nodes containing the data instead of one through the Phase series (at the level of the Study Event data in ODM), the Subject section (at the Subject level) and the sub-theme slice (at the Item Group level).

**Figure 3:**
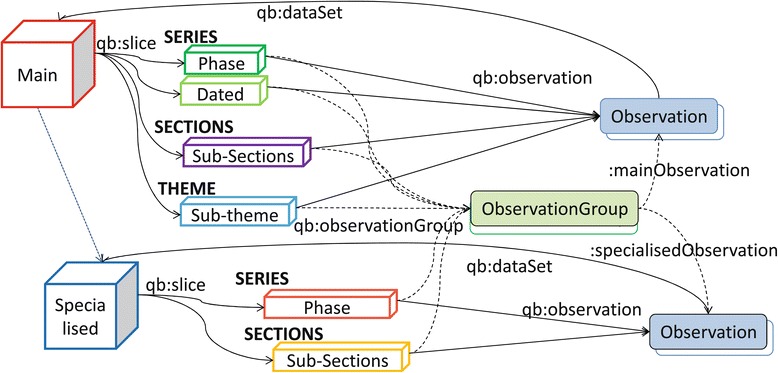
Linked clinical data cube architecture aligned with the RDF data cube. Depicts the architecture of the LCDC. The Main cube (depicted as a red cube) is split into modular Specialised cubes (depicted as a blue cube) and linked using the void:subset property. The Main cube is organised into time-series, cross-section and theme slices using the qb:slice property. The slices are then divided into Observations using the qb:observation property. The qb:dataset property is used to link the observations back to the cube. The Specialised cubes are organised similarly to the Main cube with the exception of the theme slices. The dotted lines show how the slices from all cubes interlink to the study observations through the use of ObservationGroups and the qb:observationGroup property. The mainObservation property manages the link between the ObservationGroups and the Observations while the specialisedObservation property handles the link between the ObservationGroups in the main cube and the corresponding Observations in the specialised cubes.

The slice hierarchy is provided primarily through the use of the classes and properties from the RDF Data Cube. Figure 3 highlights the LCDC slices that subsume qb:Slice. We use the void:subset property to describe the link between the main and specialised cubes. Links between slices and observations are specified using the qb:observation property, while the ones between slices and observation groups are represented by qb:observationGroup. The specialisedSeries and specialisedSection properties manage the links between the slices in the main and specialised cubes. The specialisedObservation property, which is a sub-property of qb:observation, handles the links between the observation groups from the main cube to corresponding observations in the specialised cubes.

The URI scheme describing the LCDC follows the convention from the Linked Data API [16], which uses URIs ending with an identifier to provide access to a single instance (*Item* endpoint) and URIs ending with a keyword to provide access to a list of instances (*List* endpoint).

### Phase 3: Enriching the LCDC with the CDISC standards

The CDISC CDASH and SDTM standards provide the means to standardise the clinical data. Despite not being designed around the CDISC standards, there is a good overlap between the AIBL study and the CDISC CDASH and SDTM standards for categories such as Vital Signs, Blood (represented by Laboratory Test in CDASH) and Medical History. For some categories within AIBL, the study data is clustered across many classes that do not necessarily fit to single CDASH or SDTM categories. We have chosen to map our medication data to the Concomitant Medications (CM) class within CDASH. Regarding CM, the approach taken by CDISC is to provide a framework and allow the users the ability to define the terminology of their choice. The AIBL Demographics data can be mapped to the CDISC Demographics and Subject Characteristics categories. SDTM’s Trial Arms, Trial Summary, Trial Visits and Subject Visits categories are appropriate targets for mapping longitudinal aspects of the study. For data items that are based on questionnaires, the methodology adopted by CDISC is to guide the user by providing a Questionnaire Supplements (QS) template that the user can mould to their needs. The SDTM standard provides approximately 50 questionnaires within the QS model that the user can use to model their study. The relatively low number of publicly available questionnaires is due to the fact that many of the questionnaires in clinical studies are licensed.

We have coupled the AIBL-specific variables to existing CDISC concepts, when possible, to allow a straightforward swap of variable names in a query. For example, the AIBL property for systolic blood Pressure (aiblvitalsigns:systolicBP) has been linked to the CDISC Vital Sign concept (cdiscvs:systolicBloodPressure).

### Phase 4: Mapping the data to drug terminologies

In addition to the direct coupling between AIBL and CDISC definitions described above, we have mapped the patient-reported medication intake of the AIBL participants to three external terminologies: AMT, ATC DDD^a^ and DrugBank. Our goal is to provide multiple links to hierarchical classifications of drugs. AMT provides unique codes and accurate standardised names to unambiguously identify all commonly used medicines in Australia with eight key top-level concepts [17]. We augment AMT’s capabilities with links to ARTG^b^ and UNII^c^. ARTG contains the most comprehensive list of brand names (Trade Product) in Australia, while UNII provides a non-proprietary, unambiguous and unique list of substances as maintained by the FDA. DrugBank provides a rich taxonomy of drug information alongside comprehensive drug, gene and food interactions. The appeal for our project is in the exploration of drug-drug interactions that provide some insight into the potential risks and contraindications associated with the intake of the medication. Furthermore, by exploiting the gene-drug interactions of medication targets, we can extend our framework to support the discovery of biomarkers. Finally, the availability of the food interactions will be useful when we explore the association between the participant’s drug intake and type and amount of food consumed. Both ATC DDD and DrugBank provide a supplementary means to query the data. The five-level ATC DDD taxonomy of medications provides an additional mechanism for the data to be categorised and offers the means to aggregate the study data for statistical purposes. This is complementary to what is possible with the help of the vocabularies provided by AMT.

Medication mapping is challenging due to the quality, accuracy and completeness of the information. Previous studies [8,18] have identified numerous inconsistencies linked to the naming of the medications with a mix of *trade name*, *active ingredients* and *informal name* used to describe the prescribed medications.

**Figure 4:**
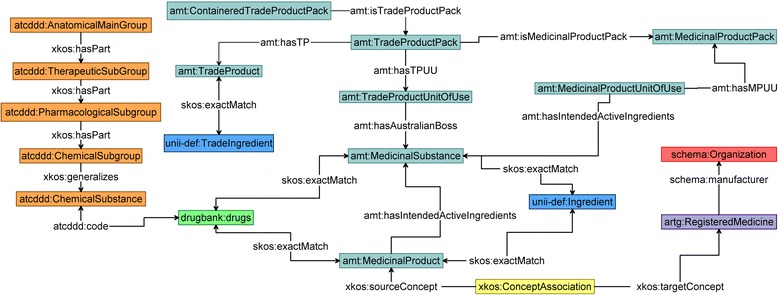
Linked Australian medications data set. Depicts the interlinking of the drug terminologies available, mostly, in Australia in order to facilitate their navigation. For the sake of simplicity, all data item variables have been omitted from the Figure. The AMT concepts are depicted in teal. The ATC DDD concepts are depicted in orange. UNII concepts are in light-blue while DrugBank concept is in light green and the ARTG concept is in magenta. The Figure also introduces an xkos:ConceptAssociation predicate (depicted in yellow) to define many-to-many relationships between amt:MedicinalProduct and artg:RegisteredMedicine concepts.

The processing pipeline for mapping the medications data to the selected medication terminologies is summarised below. The medication records are extracted from OpenClinica, at the start of the pipeline, as an XML document in CDISC ODM format. A data cleaning process is conducted to manually address the inconsistencies described above. This is followed by four mapping phases. In Phase 1, we attempt a map of the “cleaned” medication names to the Trade Product^d^ (TP) concept in AMT. We use the list of brand names compiled by ARTG to assist us in this task. In Phase 2, we try to map the same medications to the Medicinal Product^e^ (MP) concept in AMT. We use the DrugBank terms to boost the number of mapped concepts. The third phase attempts a map to the substances (active ingredients) either entered by the participants or contained within the medications recorded. To this end, we use the list provided by UNII or the Medicinal Substance^f^ (MS) defined in AMT. In Phase 4, we map the medications to the ATC DDD classification hierarchy by taking advantage of the existing mapping between the various terminologies (e.g. DrugBank and ATC DDD). We have thus compiled a linked medications data set that links AMT, DrugBank, ARTG, ATC DDD and UNII with one another as depicted in Figure 4.

## Results

The result of mapping the AIBL medications data to the medication terminologies is illustrated in Table 2. The first row discloses the total number of medications extracted. The second row represents the mappings to either a Medicinal Product, a Trade Product or a Substance in AMT. The third, fourth and fifth rows provide the mapping count for these AMT concepts individually.

**Table 2:** **Medications mapping statistics**

**Mapped**	**Count**	**Percentage**
Total	7942	100.00%
Medicinal product/trade product/substance	5536	69.71%
Trade product	5518	69.48%
Medicinal product	5266	66.31%
Substance	5382	67.77%

The Linked Clinical Data Cube has been evaluated using the full AIBL data set to demonstrate its potential in formulating queries across the broad spectrum of tests and the categories within the clinical study. While simple queries can be answered using a single data cube, more complex queries need data from several cubes to be available. The clinical data is formalised into RDF prior to being loaded in a Virtuoso triple-store.

### SPARQL Queries

To demonstrate the utility of the LCDC, we have devised a set of three questions that are typical of the questions that the AIBL researchers are likely to ask of the study data. We provide, below, a listing of the three queries. However, due to privacy constraints, we have structured our queries so that they only return aggregated counts because we are unable to present the participants unique identifier as part of the results of the queries.

Those SPARQL queries have been chosen in order to demonstrate the breadth and depth of questions that may be asked on the data set. They demonstrate how data from the AIBL study can be effortlessly combined with drug information, for example, in order to facilitate queries that answer questions based on drug classifications. Furthermore, we also demonstrate, through the integration of the AIBL data set with terminologies from the CDISC standards, how the AIBL data set can be queried by using the CDISC standardised terminology rather than the actual test names used by the AIBL study. We believe that these types of queries will drive the cross-study and cross-domain benefits of the linked clinical data approaches such as the LCDC.

#### Query 1: Using CDISC terms, find the number of participants who have hypertension

Hypertension is defined as having systolic and diastolic blood pressure readings above 140 and 90 respectively (written as 140/90 mm Hg) [19], most of the time. This query explores the use of the CDISC SDTM controlled terminology to access the diastolic and systolic blood pressure readings for participants in the AIBL study. It allows the user to interchangeably use the variable name from the AIBL study or from CDISC SDTM. 

The query obtains the relevant test names from the ontology by performing a lookup of properties that are sub-properties of the CDISC Vital Signs (prefix: vs) diastolic and systolic blood pressure variables. This is achieved by this statement:



This query is possible because we have implemented a *linked set* that connects the variable name from the AIBL study to the standardised terminology in CDISC SDTM vs domain as illustrated below.



We believe that the use of linksets in this manner is important and useful because it adheres to the principles of *information hiding* in that the user need not be aware of the exact wording of a variable. As long as the user knows the corresponding standardised variable name, the user is able to successfully execute a query on the data set. We intend to further develop this traceability mechanism with the help of the Provenance Ontology [20] to fully disclose how the published data is derived from the originally captured data.

The result of Query 1 is displayed below:

**hypertension**

242

#### Query 2: How many participants are taking an anti-diabetic drug such as Metformin?

Some studies [21,22] have shown a possible link between *type2 diabetes* and early-stage AD. In this query, we retrieve a list of anti-diabetic drugs to demonstrate the benefits of linking the patient-reported medications to standardised external terminologies and the strength of the LCDC in using federated queries to facilitate cross-domain querying. The first portion of this query obtains a list of anti-diabetic drugs from DrugBank (outlined in section A in the SPARQL). The second part of the query utilises the mappings between the patient-reported medications and DrugBank entities to link to the anti-diabetic drugs identified in section A.



The *linkset* developed to map the AMT concepts to DrugBank has been inspired from the approach described in [23,24] and uses the skos:exactMatch predicate.



The significance of this mapping is the provision of *drug-drug*, *drug-gene* and possibly *drug-disease* and *gene-gene* information relating to the AIBL study to the researchers by fully utilising the links provided by DrugBank.

The result of Query 2 is displayed in Table 3:

**Table 3:** **Participants taking anti-diabetic drugs**

**Count**	**mp_med**
1	Insulin glargine
3	Glimepiride
46	Metformin
4	Rosiglitazone
2	Glipizide
17	Gliclazide
4	Pioglitazone
1	Sitagliptin

#### Query 3: Are there participants whose classification has transitioned from healthy to mild cognitive impairment but whose triglyceride’s level has remained normal?

Research has investigated the risk factors associated with *low-density lipoproteins* or *triglycerides* on the incidence and progression of dementia and AD in later life [22]. With this in mind, we construct the query below to retrieve participants’ records whose confirmed classification status have been updated from being healthy as subjective memory complainer or non-memory complainer to having mild cognitive impairment but who have also maintained a normal (< 1.7 mmol/L) level of triglycerides in their blood sample over the course of an 18-month period between the baseline and 18-month time-points.



The above query highlights the strength of the LCDC in facilitating cross-domain queries by fully exploiting the potential of slices and observations within the specialised cubes. While the above query can be achieved without a data cube, the use of slices and observations make the query more elegant and effective. It demonstrates the navigation of the AIBL data set across two specialised cubes (Neuropsych and Blood) and four slices (two slices at each time points for each cube). These are contained within the four observations (?obs1a, ?obs1b, ?obs2a, ?obs2b) within the above query. The result of Query 3 is displayed in Table 4:

**Table 4:** **Participants’ classifications and triglycerides level**

**SubjectCount**	**Class1**	**Class2**
11	ConfirmClassification360-	ConfirmClassification360-
	memoryComplainerHealthyControl	mciPatient
4	ConfirmClassification360-	ConfirmClassification360-
	nonMemoryComplainerHealthy	mciPatient
	Control	

We provide an indication of the execution time of the three queries in Table 5 below. These queries have been executed on a Virtuoso 6.1 instance running on a virtual machine with an AMD Opteron Processor 62xx CPU, 8GB of DDR3 RAM and running Ubuntu 13.04 LTS (Raring Ringtail).

**Table 5:** **Query performances**

**Query**	**Execution time (msec)**
1	22
2	36
3	270

## Discussion and related work

Our results demonstrate the effectiveness of integrating semantic statistics vocabularies with the CDISC standards in order to expedite the navigation and querying of the data. Our contribution extends previous attempts to semantically enrich biomedical research data using ontologies [25] or linked data resources [26]. To the best of our knowledge, no study has yet investigated the association of semantic statistics vocabularies with clinical data exchange standards. The design of the LCDC was inspired by the Translational Medicine Ontology [27] and our use cases were motivated by similar objectives of providing qualitative and pertinent clinical data to the researchers and clinicians in the right format. This is what has driven our resolve to split the data into one main cube and several specialised cubes corresponding to the various domains in our study. The benefits of this approach are demonstrated in the third query where data from two specialised cubes are amalgamated to derive the results.

Observational clinical study data is patchy by nature, mainly because of the various collection mechanisms involved that often lead to information being inadvertently left out or inaccurately recorded. Furthermore, the sheer volume of variables and the longitudinal nature of the AIBL clinical study have given rise to an enormous volume of data that need to be analysed. This has led to the second design decision that is to split the data into time-series, cross-sections and themes in order to improve their manageability during the generation process and facilitate their discovery and usability by end users. Moreover, the addition of external standardised terminologies, such as the CDISC standard terminologies and the various drug vocabularies utilised, have contributed not only to standardising the data and to removing ambiguities but to enriching the data by providing links to relevant online resources, such as genes and pathways definitions and information about their interactions with the entities.

### Challenges in the use of the CDISC standards as the underlying model

While the CDISC models suit our immediate purpose, they present a few shortcomings, mainly in relation to the semantics associated with the clinical study data. ODM’s constrained hierarchical structure largely promotes single-study explorations of clinical study data. Furthermore, the inability to store domain information alongside the user-defined data items in the customisable CRFs is, in our view, very restrictive, thus impeding their use outside of the study context [28]. However, this stems more from the various failings in the implementation of the CDISC standards by the vendors. The ODM standard allows for CDASH terms to be inserted through the use of annotations within the ODM XML model. However, several vendors, such as OpenClinica, choose not to offer this feature natively within their tool. Abler et al. [28] make a passionate claim for the definition of a *language of forms* that can effectively record the *logical relationships between questions or sets of questions* asked in the forms.

On a more technical aspect, ODM also suffers from a lack of established complex data type standards, thus allowing a study coordinator to provide an alternative definition for, say, the *Physical Quantity* data type. Furthermore, despite the provision of detailed *Implementation Guides* describing the correct way of encoding data items, the definition of very coarsely granulated meta-data categories, such as *Medical History* in SDTM, opens up the possibility, for the user, to capture semantically identical data in multiple domains. While the lack of data standards is a problem, the lack of mechanisms to enforce adherence to these data standards is a greater problem. As such, despite CDISC providing mechanisms, through its SDTM and CDASH models, to define common semantics, in our experience, very few study coordinators choose to use them.

Our choice of the CDISC standard as the underlying model for our architecture is influenced by three factors: (i) since the FDA and other regulatory bodies mandate the use of CDISC as the *de facto* standards for representing and reporting clinical study data, a vast majority of the clinical study data that we encounter is already in CDISC format; (ii) several extensions to the CDISC standards (such as the *Therapeutic Area* standard for Alzheimer’s Disease) are appealing to us; and (iii) we have *not yet* found a consistent and complete set of ontologies that we could use instead.

In our approach to semantically enrich the clinical study data, we need to address the study-specific nature of CDISC ODM datasets. We inherit many issues that have been created in the previous steps of the data capture chain, such as the use of user-defined questionnaires and instruments that use their own language and the loss of domain knowledge during the digitisation phase of the data. Our solution is to reintroduce the loss of domain-specific information by first trying to retrofit the study variables to the SDTM and CDASH models, even though they were not initially modelled that way. Concurrently, we look to biomedical ontologies, such as the NCBO Bioportal ontologies [29] and SNOMED CT^g^, to provide alternative foundations for domain enrichment of the data set. Several ontologies, in the context of clinical trials [30-32], have been proposed recently and are partially applicable to our needs. However, they do not adequately cover the observational aspects that are required for our data cubes. Furthermore, several of these ontologies have a large number of dependencies to other ontologies that do not meet our requirements. We overcome the limitations related to the single-study nature of ODM by fitting the study data to the RDF Data Cube. The introduction of additional dimensions, through the integration to the RDF Data Cube, opens up new access points to the data through the use of the thematic slices.

Ultimately, our view is that regulatory bodies have a pivotal role to play in encouraging the clinical study coordinators to engage with data scientists at an earlier stage in their clinical study to help with the design of their study and associated artefacts. Too much emphasis is placed on the data collection phase and not enough effort is expended in clarifying what is needed to analyse the data.

### Related work

The Linked Open Drug Data (LODD) [33] and the Linked Life Data (LLD) [34] projects provide additional resources that can be used to extend the Linked Clinical Data Cube. Both projects aim to build a large scale knowledge cloud that can be used for drug discovery. LODD federates the efforts by participants of the W3C Semantic Web Health Care and Life Sciences (HCLS) Interest group to convert available resources into linked data. LLD provides a semantic data integration platform for the biomedical domain comprising many of the data sources belonging to LODD. The resulting datasets contains more than 8 million triples representing the knowledge within over 2 millions links relating to medications, diseases, clinical trials, gene information and pharmaceutical companies among others. This was followed by efforts to convert the ChEMBL database as linked open data [23]. This new linked dataset combines the description of the biological entities with links to *Bio2RDF* [35], *ChemSpider* [36], *OpenMoleculesRDF* [37] and *CrossRef* [38] to allow dereferenceable access to a myriad of external datasets. We have adopted a similar methodology in our approach to map the medications specialised cube to AMT, DrugBank and ATC DDD.

Among the various use cases reported via the W3C HCLS Interest group are efforts to explore links to identify and verify genes linked to Alzheimer’s disease (AD). Through the links between the drug, medications, disease and clinical trial repositories, we hope to leverage on efforts by others to further explore the effects of prescribed medications, for AD sufferers, on the various genes comprising the pathways of interest. Other applications of LODD include the identification of potential side-effects linked to the intake of drugs that have conflicting stimuli on the disease pathways.

The SALUS project [39] is a former attempt to adapt CDISC standards to build a Semantic Framework to improve interoperability between clinical research and clinical care domains. We adopt a similar approach to them but their focus is on service mappings rather than linked data sets. The Semantic Cockpit [40] project aims to develop a data slicing framework comparable to what we propose on the basis of the RDF Data Cube. The goal of this project is to intelligently assist business analysts by discriminating unimportant information and using reasoning to only present useful information to the analyst.

The *Linked Medical Data Access Control* (LiMDAC) project [41] has devised a framework to enable the integration of medical data without compromising its privacy, security and integrity. It defines three linked data models that use the RDF Data Cube to build an access control framework that restricts access to the aggregated data.

The *Pharmaceutical Users Software Exchange* [42] community, in concert with the FDA, has started work on RDF representations of various CDISC models [43], including the terminologies published by the *National Cancer Institute (NCI) Enterprise Vocabulary Services* [44]. This community has started to evaluate the RDF Data Cube [45,46] for the publication of clinical study data. These conversions of comma-separated-value files, however, do not fully exploit the relationships between the data and metadata structures embedded within the XML versions of the CDISC standards and the patterns and concept definitions included in the generated RDF content.

## Conclusions

This paper has outlined the semantic enrichment of longitudinal clinical study data based on the CDISC standards with elements from the semantic statistics vocabularies, namely the RDF Data Cube and the DDI-RDF Discovery vocabularies. We have outlined how the Health Care and Life Science community is likely to benefit from the adoption of tools and techniques that will deliver clinical data as linked data and advance its integration with complementary data sources. In this regard, we have proposed a Linked Clinical Data Cube, which integrates one main and several specialised data cubes to provide increased flexibility in the navigation of the clinical data and allow the users to formulate the queries more efficiently and effectively. The Linked Clinical Data Cube combines the strength of the RDF Data Cube in defining multi-dimensional data cubes and the DDI-RDF Discovery vocabulary in encoding the CDISC metadata and the study specific data dictionary as linked data. Our approach was validated using data captured as part of a longitudinal clinical study into neurodegenerative diseases. This research has resulted in four contributions. First, we have uncovered the complementarities of the RDF Data Cube and DDI-RDF Discovery vocabularies for the publication of large heterogeneous data sets as linked data. Second, we have demonstrated the fit of the semantic statistics vocabularies to enrich the CDISC ODM data model for the publication of clinical study data as linked data. Third, we have illustrated how the clinical study data has been semantically enriched with links to external resources and how they ultimately improve the navigation and querying of the data. Fourth, we have built the foundations of a framework supporting cross-domains and cross-study analysis by adopting a more standardised data structure. Our next step is to enrich the remaining study data set with concepts from other domain ontologies, such as Blood, Neuropsychological tests and Nutrition, to name just three.

## Endnotes

^a^ Anatomical Therapeutic Chemical Defined Daily Dose.

^b^ Australian Register of Therapeutic Goods.

^c^ Unique Ingredient Identifier.

^d^30560011000036108 | trade product |.

^e^30497011000036103 | medicinal product|.

^f^30388011000036105 | medicinal substance |.

^g^ Systematized Nomenclature of Medicine Clinical Terms.
